# Regional pancreatoduodenectomy *versus* standard pancreatoduodenectomy with portal vein resection for pancreatic ductal adenocarcinoma with portal vein invasion

**DOI:** 10.1002/bjs5.50268

**Published:** 2020-03-19

**Authors:** A. Oba, H. Ito, Y. Ono, T. Sato, Y. Mise, Y. Inoue, Y. Takahashi, A. Saiura

**Affiliations:** ^1^ Division of Hepatobiliary and Pancreatic Surgery Cancer Institute Hospital, Japanese Foundation for Cancer Research, 3‐8‐31 Ariake, Koto‐ku Tokyo 135‐8550 Japan

## Abstract

**Background:**

Pancreatoduodenectomy (PD) with portal vein resection (PVR) is a standard operation for pancreatic ductal adenocarcinoma (PDAC) with portal vein (PV) invasion, but positive margin rates remain high. It was hypothesized that regional pancreatoduodenectomy (RPD), in which soft tissue around the PV is resected *en bloc*, could enhance oncological clearance and survival.

**Methods:**

This retrospective study included consecutive patients who underwent PD with PVR between January 2005 and December 2016 in a single high‐volume centre. In standard PD (SPD) with PVR, the PV was skeletonized and the surrounding soft tissue dissected. In RPD, the retropancreatic segment of the PV was resected *en bloc* with its surrounding soft tissue. The extent of lymphadenectomy was similar between the procedures.

**Results:**

A total of 268 patients were included (177 SPD, 91 RPD). Tumours were more often resectable in patients undergoing SPD (60·5 per cent *versus* 38 per cent in those having RPD; *P* = 0·014), and consequently they received neoadjuvant therapy less often (7·9 *versus* 25 per cent respectively; *P* < 0·001). R0 resection was achieved in 73 patients (80 per cent) in the RPD group, compared with 117 (66·1 per cent) of those in the SPD group (*P* = 0·016), although perioperative outcomes were comparable between the groups. Median recurrence‐free (RFS) and overall (OS) survival were 17 and 32 months respectively in patients who had RPD, compared with 11 and 21 months in those who had SPD (RFS: *P* = 0·003; OS: *P* = 0·004).

**Conclusion:**

RPD is as safe and feasible as SPD, and may increase the survival of patients with PDAC with PV invasion.

## Graphical abstract

A regional pancreatoduodenectomy (RPD) procedure was employed to enhance oncological clearance around the mesenteric vein in patients with locally advanced pancreatic cancer. This provided better local control with longer overall survival than a standard pancreatoduodenectomy procedure.

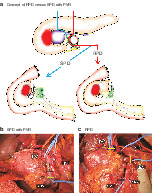

Regional pancreatectomy associated with increased R0‐rate and survival

## Abstract (es)

**Antecedentes:**

La duodenopancreatectomía (*pancreaticoduodenectomy*, PD) con resección de la vena porta (*portal vein resection*, PVR) es una operación estándar para el adenocarcinoma ductal pancreático (*pancreatic ductal adenocarcinoma*, PDAC) con invasión de la vena porta (*portal vein*, PV); sin embargo, las tasas de margen positivo siguen siendo altas. Nuestra hipótesis fue que la duodenopancreatectomía regional (*regional pancreaticoduodenectomy*, RPD) en la que el tejido blando alrededor de la PV se reseca en bloque podría mejorar el resultado oncológico y la supervivencia.

**Métodos:**

Este estudio retrospectivo incluyó pacientes consecutivos que se sometieron a PD con PVR entre enero de 2005 y diciembre de 2016 en un solo centro de alto volumen. En la PD estándar (SPD) con PVR, la PV se esqueletizó disecando el tejido blando circundante. En la RPD, el segmento retropancreático de la PV se resecó en bloque con el tejido blando circundante. La extensión de la linfadenectomía fue similar en ambos procedimientos.

**Resultados:**

Se incluyeron un total de 268 pacientes (177 sometidos a SPD y 91 a RPD). Los pacientes sometidos a SPD presentaron con mayor frecuencia tumores resecables (35 (38%) *versus* 107 (61%), *P* = 0,014)) y recibieron con mayor frecuencia terapia neoadyuvante (23 (25%) *versus* 14 (8%), *P* < 0,001)) que los pacientes sometidos a RPD.

La resección R0 se logró en 73 (80%) pacientes pertenecientes al grupo RPD, en comparación con 117 (66%) pacientes sometidos a SPD (*P* = 0.011), aunque los resultados perioperatorios fueron comparables entre los grupos. La mediana de supervivencia libre de recidiva (*recurrence‐free survival*, RFS) y de supervivencia global (*overall survival*, OS) fueron 17 meses y 31 meses, respectivamente, en pacientes sometidos a RPD, en comparación con 11 meses y 21 meses en pacientes sometidos a SPD, (*P* = 0,003 para RFS y *P* = 0,004 para la OS).

**Conclusión:**

La RPD es tan segura y factible como la SPD y puede aumentar la supervivencia de pacientes con PDAC con invasión de la PV.

## Introduction

Complete resection is considered a cornerstone in curative therapy for pancreatic cancer, but sufficient margin clearance during pancreatoduodenectomy (PD) for pancreatic ductal adenocarcinoma (PDAC) has remained challenging because of the proximity to mesenteric vessels that must be preserved: the portal vein (PV), superior mesenteric vein (SMV) and artery (SMA). Although concomitant PV/SMV resection has been adopted widely and has improved the resectability of PDAC with suspected PV invasion, the positive resection margin rate remains high, ranging from 22 to 63 per cent^1^, ^2^, ^3^, ^4^, most commonly for the posteromedial margin near the SMV and SMA^3^. Clinically, patients with PDAC have a poorer long‐term outcome after PD with positive resection margins than after PD with negative margins^5^. Therefore, increasing the length of the resection margin near the mesenteric vessels during PD is critical to enhance oncological clearance and improve the prognosis in patients with locally advanced PDAC undergoing vascular invasion.

In 1973, Fortner and colleagues^6^ described a novel pancreatic resection that they named regional pancreatectomy (RP). This radical operation was characterized by routine portomesentericosplenic confluence (PMSC) resection *en bloc* with the surrounding soft tissue (type I), in contrast to conventional PD with PV resection (PVR), in which the resected vessel is skeletonized except for the portion with tumour invasion in order to minimize the length of PV to be resected^7^. Although Fortner's RP procedure was not accepted widely because of associated morbidity, the surgical concept remains attractive in terms of increasing the length of the margin and thereby increasing the chance of R0 resection for PDAC with potential invasion to the PV/SMV. The present authors therefore hypothesized that regional pancreatoduodenectomy (RPD) could improve patient outcomes in the modern era of sophisticated perioperative management and effective systemic chemotherapy.

This concept was adopted into the authors' contemporary pancreatoduodenectomy with the SMA‐first approach, and a new surgical procedure was designed for PD. The new RPD approach is simpler and less radical than the original RP, involving no total pancreatectomy, no arterial resection and no extended lymphadenectomy. This study aimed to describe RPD and address its short‐ and long‐term outcomes compared with those for standard PD (SPD) with PVR in patients with advanced PDAC.

## Methods

The Cancer Institute Hospital Institutional Review Board approved the study. A retrospective review was undertaken of the medical records of consecutive patients who had PD for pancreatic adenocarcinoma from January 2005 to December 2016, and patients who underwent PD with PVR were included.

The authors' approach to the management of pancreatic cancer has been described previously^8^. In brief, all presenting patients are evaluated with preoperative imaging studies including thin multidetector CT of the chest, abdomen and pelvis, and MRI of the liver for preoperative staging. Resectability is determined before surgery based on the CT findings according to National Comprehensive Cancer Network (NCCN) guidelines^9^. After January 2015, all patients with borderline resectable tumours received neoadjuvant chemotherapy with gemcitabine and nab‐paclitaxel, followed by surgical resection. Before that period, patients with resectable or borderline resectable tumours had upfront surgery. After successful postoperative recovery, patients received adjuvant chemotherapy with gemcitabine or S1, and were followed with serial CT and blood tests every 3 months. No patient received radiotherapy in either the adjuvant or neoadjuvant setting.

### Surgical techniques: regional PD *versus* standard PD with portal vein resection

The indication for PVR was determined based on preoperative CT findings: when direct contact of the tumour to the PV was suspected, PVR was planned. The type of resection (SPD *versus* RPD) was chosen at the surgeon's discretion. The technical detail of the authors' standard PD (supracolic anterior artery‐first approach) has been described previously^10^.

In SPD, the SMA is dissected first, and the inferior pancreatoduodenal artery (IPDA) is ligated and divided before division of the pancreas along the line above or to the left of the PV/SMV. The soft tissue around the retropancreatic PV/SMV is then dissected and the PV/SMV skeletonized to minimize the length of vessel resection. The PMSC is preserved when possible, and resected only when in extensive contact with tumour.

**Figure 1:**
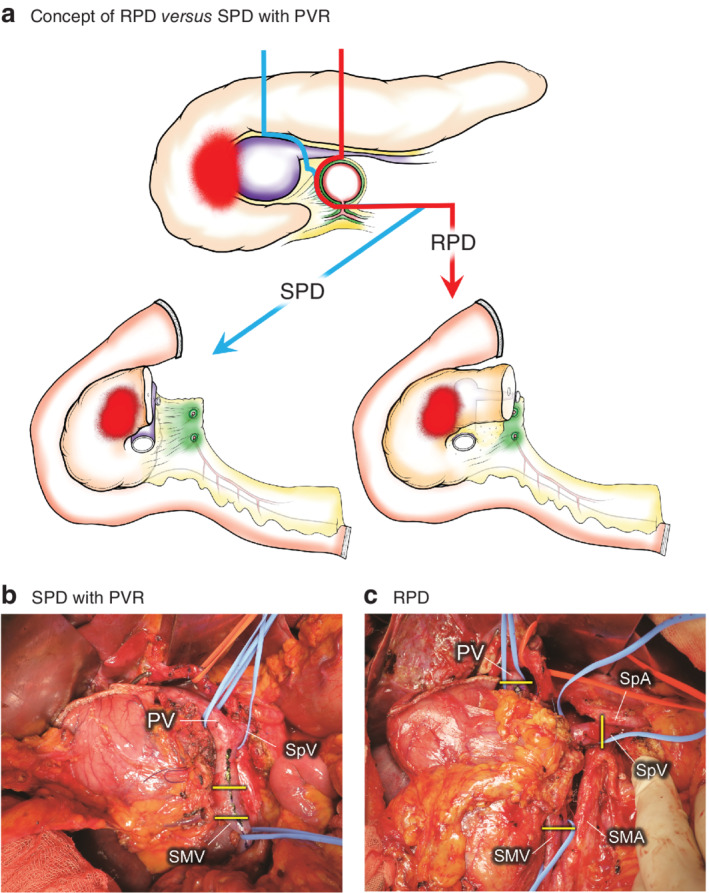
Comparison of regional pancreatoduodenectomy and standard pancreatoduodenectomy with portal vein resection **a** Concept of regional pancreatoduodenectomy (RPD) compared with standard pancreatoduodenectomy (SPD) with portal vein (PV) resection (PVR). Although all soft tissue around retropancreatic segments of the PV is removed *en bloc* in RPD, the PV and superior mesenteric vein (SMV) are skeletonized except at the site of tumour invasion; thus dissected soft tissue, potentially containing residual cancer cells, could be left behind in SPD. In the resected specimen after RPD, retropancreatic PV is covered circumferentially by soft tissue and should not be visible. Note that the depth of superior mesenteric artery (SMA) dissection is no different in the two procedures. **b,c** Representative intraoperative photographs at the last step of resection. The retropancreatic segment of PV/SMV is covered by fatty soft tissue and is not visible in RPD (**c**). In contrast, almost the entire length of the PV/SMV, including the splenic vein (SpV) junction, is skeletonized in SPD with PVR (**b**). The yellow lines indicate the line of division of mesenteric veins. SpA, splenic artery.

In RPD, the retropancreatic segment of the PV/SMV/PMSC and surrounding soft tissue are left untouched, and the pancreas is divided along the line above the SMA (*Fig*. 1). The PV and SMV are encircled superiorly and inferiorly to the pancreatic head, and the splenic vein (SpV) above the SMA. These vessels are divided at the final step of resection away from the tumour. The PV/SMV stumps are anastomosed primarily and an interposition graft is rarely used. The right colon is fully mobilized so that the vein ends can be apposed without tension when the gap is long. The SpV stump is either simply ligated or reconstructed with anastomosis to the outflow vessels including the PV/SMV, left renal vein, middle colic vein, gonadal vein and inferior mesenteric vein at the surgeon's discretion^11^, ^12^. All other components of the RPD procedure are as described for the SPD, with no differences in the depth of SMA dissection or extent of lymphadenectomy.

The first RPD was performed by the authors' group in 2008, and subsequently both procedures (RPD and SPD) have been performed according to surgeon preference. A representative video of RPD is provided as *Video* *S1* (supporting information).

### Pathological evaluation and follow‐up for recurrence

The pathologist handled all resected specimens according to the standard procedure; margin status was reported separately for the pancreatic stump, bile duct stump and dissected peripancreatic tissue. When the dissected peripancreatic tissue margin was positive, the location was described in detail. The orientation for each margin in the resected specimens was presented and verified at a weekly hepatopancreatobiliary pathology conference attended by surgeons and pathologists.

A microscopically positive margin (R1) was defined by the presence of cancer cell in any margin, and R0 was reported when no cancer cells were detected in the margins, regardless of the distance between the tumour and the closed margin.

Recurrence of the disease after resection was judged primarily by CT with intravenous contrast every 3–6 months after surgery, supplemented selectively by MRI and PET. Patients were usually prescribed systemic chemotherapy without histological confirmation by biopsy when CT and other clinical findings indicated disease recurrence.

Local recurrence was defined as recurrence near the previous surgical bed or in the regional lymph nodes, and distant recurrence was defined as recurrence in the distant organs, including liver, lung and peritoneum.

Data were retrieved retrospectively and analysed from medical records, including surgical and pathology reports, as follows: patient demographics; preoperative tumour characteristics including size and resectability based on NCCN guidelines^9^; pathological features including T and N category, number of harvested lymph nodes and margin status; postoperative variables including use of adjuvant chemotherapy, and the date of recurrence or death. The final pathological stage was based on the eighth edition of the UICC TNM classification^13^. Classification of type of surgical approach (RPD or SPD) was verified based both on the surgical report documented by the operating surgeon and on images of the final specimens. When the surface of resected retropancreatic segments of the PV/SMV was exposed in the surgical specimen, the operation was not considered as RPD.

Recurrence‐free (RFS) and overall (OS) survival were determined from the time of resection to the time of detection of first recurrence and death or last follow‐up respectively. The dates for detection of initial local recurrence and distant recurrence were recorded separately.

### Statistical analysis

Statistical analyses were performed using SPSS® version 24.0 (IBM, Armonk, New York, USA). Continuous variables are expressed as median (range) values and compared with the Mann–Whitney *U* test, and categorical variables are compared with the χ^2^ or Fisher's exact test. Survival curves were constructed using Kaplan–Meier methods and compared with the log rank test. Potential prognostic variables were evaluated by multivariable analysis using Cox regression. Two‐tailed *P* < 0·050 was considered statistically significant.

## Results

Of 431 patients with PDAC who underwent PD during the study period, 268 (62·2 per cent) had *en bloc* PVR and were included in the study. Ninety‐one patients (34·0 per cent) underwent RPD and the rest had SPD with PVR. The demographics of the cohort patients and the tumour characteristics are summarized in *Table* 1. There was no difference in age or sex between the groups, but tumours in the RPD group were more advanced than those in the SPD group in terms of resectability, as graded by preoperative CT (borderline resectable or unresectable: 62 *versus* 39·5 per cent respectively, *P* = 0·014). As a result, more patients in the RPD group received neoadjuvant chemotherapy than those in the SPD group (25 *versus* 7·8 per cent respectively; *P* < 0·001). Most patients did not receive neoadjuvant therapy as routine use of preoperative chemotherapy for borderline resectable tumours was initiated in 2015.

**Table 1:** Patient demographics and tumour characteristics

	RPD (*n* = 91)	SPD with PVR (*n* = 177)	*P* ‡
**Age (years)** *	68 (47–86)	67 (37–86)	0·639§
**Sex ratio (M** : **F)**	43 : 48	97 : 80	0·248
**BMI (kg/m** ^**2**^ **)** *	21·8 (16–46)	21·7 (16–30)	0·848§
**Preoperative CEA level (ng/ml)** *	3 (0·5–36)	3 (0·5–44)	0·624§
**Preoperative CA19‐9 level (units/ml)** *	60 (2–50 000)	187 (2–24 464)	0·527§
**Preoperative chemotherapy**			< 0·001
Yes	23 (25)	14 (7·9)	
No	68 (75)	163 (92·1)	
**Adjuvant chemotherapy**			0·354
Yes	74 (81)	134 (75·7)	
No	17 (19)	43 (24·3)	
**Resectability** †			0·014
Resectable	35 (38)	107 (60·5)	
Borderline resectable	53 (58)	67 (37·9)	
Uunresectable	3 (3)	3 (1·7)	
**Tumour size (cm)** *	3·5 (1·3–7·1)	3·5 (1·3–9·1)	0·513§
**pT category**			0·262
pT1	9 (10)	9 (5·1)	
pT2	53 (58)	101 (57·1)	
pT3	29 (32)	67 (37·9)	
pT4	0 (0)	0 (0)	
**pN category**			0·445
pN0	26 (29)	40 (22·6)	
pN1	38 (42)	73 (41·2)	
pN2	27 (30)	64 (36·2)	
**Treatment years**			< 0·001
2005–2010	13 (14)	73 (41·2)	
2011–2016	78 (86)	104 (58·8)	

Values in parentheses are percentages unless indicated otherwise

* values are median (range).

† Based on the CT findings at initial presentation. RPD, regional pancreatoduodenectomy; SPD, standard pancreatoduodenectomy; PVR, portal vein resection; CEA, carcinoembryonic antigen; CA, carbohydrate antigen.

‡ χ^2^ or Fisher's exact test, except

§ Mann–Whitney *U* test.

### Short‐term outcomes for regional *versus* standard pancreatoduodenectomy

*Table* 2 summarizes the short‐term surgical outcomes. There was no difference in duration of surgery or blood loss between the RPD and SPD groups. Regarding PVR, 57·1 per cent of patients who underwent SPD had the SpV preserved, whereas it was resected in all patients who had RPD. The length of resected PV was significantly greater for RPD procedures than for standard PD (median 4 *versus* 3 cm respectively). Postoperative morbidity of Clavien–Dindo grade IIIa or above was similar regardless of procedure, and only two patients died from surgical complications, following SPD.

**Table 2:** Surgical details and perioperative outcomes

	RPD (*n* = 91)	SPD with PVR (*n* = 177)	*P* †
**Division line of pancreas**			< 0·001
Above PV	0 (0)	78 (44·1)	
Above SMA	47 (52)	83 (46·9)	
Above SpA	44 (48)	16 (9·0)	
**Type of PVR**			< 0·001
PMSC resection	91 (100)	76 (42·9)	
PV resection	0 (0)	90 (50·8)	
SMV resection	0 (0)	11 (6·2)	
**Type of PV reconstruction**			< 0·001
Primary anastomosis	90 (99)	137 (77·4)	
Interposition graft	1 (1)	9 (5·1)	
Wedge resection with patch	0 (0)	31 (17·5)	
**Length of resected vein (cm)** *	4 (2–8)	3 (1–6)	< 0·001‡
**No. of retrieved lymph nodes** *	39 (12–87)	35 (3–80)	0.081‡
**No. of positive lymph nodes** *	2 (0–23)	2 (0–20)	0.550‡
**Overall surgical margin status**			0·016
R0	73 (80)	117 (66·1)	
R1	17 (19)	57 (32·2)	
R2	1 (1)	3 (1·7)	
**No. of positive medial margins**	12 (13)	50 (28·2)	0·006
**Duration of surgery (min)** *	528 (370–989)	545 (363–1118)	0.165‡
**Intraoperative blood loss (ml)** *	660 (50–2400)	700 (80–6700)	0.194‡
**Transfusion**	9 (10)	35 (19·8)	0.054
**Clavien–Dindo complications**			0.349
Grade 0–II	79 (87)	155 (87·6)	
Grade III	12 (13)	17 (9·6)	
Intra‐abdominal bleeding	3	2	
Intra‐abdominal abscess	1	5	
Pancreatic fistula	1	5	
Diarrhoea	1	0	
Ascites	2	2	
Bile leak	1	1	
Bowel obstruction	1	0	
Delayed gastric emptying	1	1	
Cholangitis	0	1	
Acute renal failure	1	0	
Grade IV	0	3 (1·7)	
Sepsis	0	2	
Pneumonia	0	1	
Grade V	0	2 (1·1)	
Intra‐abdominal bleeding	0	1	
Sepsis	0	1	
**Postoperative mortality**	0	2 (1·1)	0.550

Values in parentheses are percentages unless indicated otherwise

* values are median (range). RPD, regional pancreatoduodenectomy; SPD, standard pancreatoduodenectomy; PVR, portal vein resection; PV, portal vein; SMA, superior mesenteric artery; SpA, splenic artery; PMSC, portomesentericosplenic confluence; SMV, superior mesenteric vein.

† χ^2^ or Fisher's exact test, except

‡ Mann–Whitney *U* test.

In terms of oncological outcomes, RPD was significantly less likely than SPD to result in positive resection margins, despite the similar median size of resected tumours (overall positive margin rate: 20 *versus* 33·9 per cent respectively, *P* = 0·016; positive medial margin rate: 13 *versus* 28·2 per cent, *P* = 0·006) (*Table* 2). As the extent of lymphadenectomy was similar for RPD and SPD, there was no difference in the number of retrieved lymph nodes between the two procedures (median 39 for RPD and 35 for SPD; *P* = 0·081).

### Impact of regional PD on long‐term outcomes in advanced disease

The median duration of follow‐up was 24 (range 1–163) months across the entire cohort and 38 (16–163) months in survivors. During follow‐up, 23 patients (25 per cent) who had RPD developed documented local recurrence, compared with 67 (37·9 per cent) who underwent SPD with PVR (*P* = 0·039).

**Figure 2:**
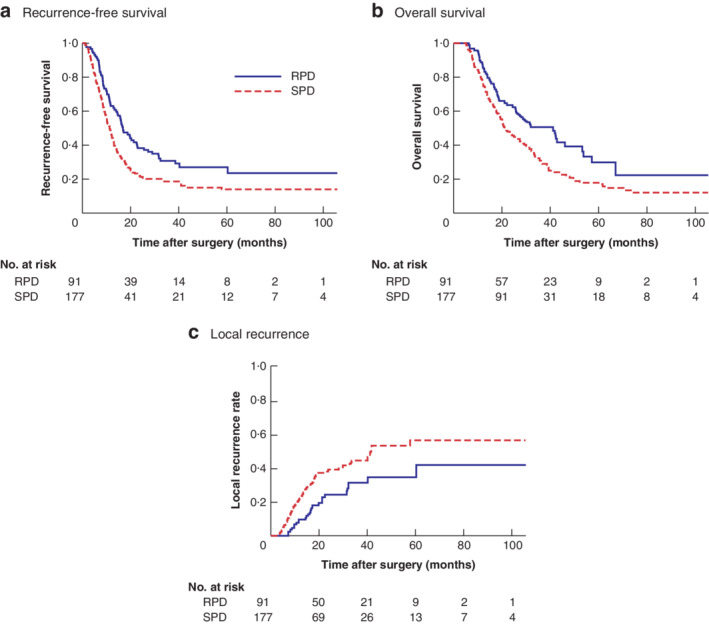
**Kaplan–Meier analysis of recurrence‐free and overall survival, and local recurrence in patients with pancreatic duct adenocarcinoma who had regional pancreatoduodenectomy or standard pancreatoduodenectomy with portal vein resection** **a** Recurrence‐free survival, **b** overall survival and **c** local recurrence. RPD, regional pancreatoduodenectomy; SPD, standard pancreatoduodenectomy. **a** *P* = 0·003, **b** *P* = 0·004, **c** *P* = 0·012 (log rank test).

*Fig*. 2 shows Kaplan–Meier curves for RFS and OS among all patients. Median RFS and OS after RPD was 17 and 32 months respectively, compared with 11 and 21 months after SPD (RFS: *P* = 0·003; OS: *P* = 0·004).

Univariable and multivariable analyses of predictive variables for RFS and OS following resection are presented in *Table* 3. Four patients who had resection with macroscopic residual disease (R2) were excluded from these analyses. RPD was one of the variables identified as an independent determinant for both RFS and OS; other such variables included carbohydrate antigen 19‐9, R status, lymph node metastasis and use of adjuvant therapy.

**Table 3:** Univariable and multivariable analysis of factors associated with recurrence and overall survival after R0–1 resection

		Recurrence‐free survival				Overall survival			
	*n*	Median (months)	Univariable *P*	Multivariable *P*	HR	Median (months)	Univariable *P*	Multivariable *P*	HR
**Preoperative factors**									
CA19‐9 (units/ml)									
> 200	116	10	< 0·001	0·006	1·50 (1·1, 2·0)	18	< 0·001	0·004	1·50 (1·1, 2·0)
≤ 200	148	14				30			
Radiographic resectability									
Resectable	138	13	0·338			24	0.294		
Borderline or unresectable	126	11				21			
**Tumour‐related factors**									
pT category									
pT1–2	170	14	0·032	0·389		27	0·019	0.280	
pT3	94	11				19			
pN category									
pN0	66	22	< 0·001	0·001	0·74 (0·62, 0·89)	37	0·001	0·008	0·63 (0·44, 0·89)
pN1–2	198	11				20			
Tumour differentiation									
Well or moderate	226	13	0·323			26	0·354		
Poor	38	12				19			
Histological PV invasion									
Yes	114	11	0·164			20	0·276		
No	150	13				28			
Margin status									
R0	190	13	< 0·001	0·023	0·84 (0·72, 0·98)	28	0·001	0·001	0·61 (0·45, 0·82)
R1	74	10				17			
**Treatment factors**									
Type of resection									
RPD	90	17	0·003	0·018	0·83 (0·71, 0·97)	32	0·004	0·029	0·70 (0·51, 0·96)
SPV with PVR	174	11				20			
Use of preoperative chemotherapy									
Yes	37	17	0·036	0·571		46	0·032	0·676	
No	227	11				21			
Use of adjuvant chemotherapy									
Yes	206	13	< 0·001	0·002	0·76 (0·64, 0·91)	29	< 0·001	< 0·001	0·43 (0·31, 0·60)
No	58	8				13			

Values in parentheses are 95 per cent confidence intervals. Patients who had an R2 resection were excluded from the analysis. HR, hazard ratio; CA, carbohydrate antigen; PV; portal vein; RPD, regional pancreatoduodenectomy; SPD, standard pancreatoduodenectomy; PVR, portal vein resection.

## Discussion

This study from a high‐volume cancer centre compared two surgical approaches for PDAC with PV invasion: RPD in which retropancreatic segments of the PV, SMV and SpV were resected *en bloc* with surrounding soft tissue, and SPD in which the PV was skeletonized and resected only at the site of tumour invasion. RPD was safe and feasible with comparable surgical morbidity to SPD. Although there has been an ongoing debate about the role of radical surgical procedures for PDAC, this is the largest series among the few studies suggesting improved long‐term outcomes for RPD compared with SPD for patients with PDAC with PV invasion.

It is important to acknowledge that this study is not a simple comparison of radical and non‐radical surgical procedures. Although the surgical safety of concomitant PVR during PD has been shown in many series, a meta‐analysis by Giovinazzo and co‐workers^14^ including more than 1500 patients who had PD with PVR for pancreatic cancer demonstrated a higher postoperative morbidity rate (39 per cent) and higher chance of incomplete resection (R1–2, 37 per cent) after PD with PVR, compared with 32 and 31 per cent respectively after PD without PVR (morbidity: *P* = 0·03; incomplete resection: *P* < 0·001). Thus, PVR alone would add significant complexity and morbidity to the already complex pancreatic resection, and the surgical indication therefore needs to be selected carefully based on the surgeon's experience and skills following precise evaluation of the disease extent by preoperative imaging. The present authors perform PVR only when there is evidence of direct contact between the tumour and the PV on preoperative CT. Indeed, more than 60 per cent of the present PD procedures for PDAC were done with concomitant PVR. However, when the tumour to SMA contact exceeds 180°, the authors consider the tumour unresectable and no pancreatic resection is planned unless the tumour shrinks significantly with chemotherapy.

According to the original Fortner report^6^, three of 18 patients who underwent RP died within 30 days of surgery, and two more died from complications after 30 days, giving an operative mortality of 28 per cent. In contrast, the 90‐day mortality rate among the present 91 patients who had RPD was zero, and severe complications of Clavien–Dindo grade IIIb or above occurred in only 12 patients (13 per cent). RPD would not expand the current resectability criteria for otherwise unresectable locally advanced PDAC. In contrast to Fortner's approach, arterial resection to achieve negative margins around the SMA was not attempted, and the present authors considered any arterial involvement as a definitive contraindication to resection. Indeed, data specific for the outcomes for PD with SMA resection are limited in the current literature^15^, ^16^. Although multiple small case series^17^, ^18^, ^19^ from other high‐volume centres reveal an acceptable surgical morbidity rate for PD with SMA resection, it remains unclear whether this vascular resection and reconstruction could improve the patient's long‐term survival. Instead of SMA resection, the present authors excised *en bloc* the right side of the periarterial neural plexus around the SMA when perioperative CT showed tumour to SMA contact of less than 180°^10^, ^20^ and, because of concern for intractable diarrhoea caused by overly aggressive neural dissection with no proven survival advantage^21^, ^22^, ^23^, they do not recommend routine dissection around the SMA.

Although lymph node metastasis was a significant prognostic factor in many studies^1^, ^4^, ^19^, ^24^, including the present study, the optimal extent of lymphadenectomy for PDAC remains to be defined. Fortner^25^ performed very wide ‘regional’ lymphadenectomy, including the retroperitoneal para‐aortic and coeliac axis lymph nodes, in their original RP. In contrast, the present authors limited the extent of lymphadenectomy by excluding these node stations. Indeed, among the five RCTs^22^, ^23^, ^26^, ^27^, ^28^ conducted to determine the optimal extent of lymphadenectomy, none involving ‘extended’ lymphadenectomy demonstrated improved survival for patients over those involving the ‘standard’ one. Furthermore, in the meta‐analysis reported by Orci and colleagues^29^ extended lymphadenectomy was associated with increased duration of surgery, blood loss and morbidity.

It should be noted that both SPD and RPD described in this report are variants of the ‘artery‐first’ PD in which SMA dissection with IPDA division is completed before the pancreatic division. As shown previously^10^, this artery‐first approach reduces intraoperative blood loss compared with conventional ‘artery‐last’ PD. Strasberg *et al*.^30^ described an extended PD with PMSC resection for PDAC with extensive PV/SMV invasion, performed with no surgical mortality in ten patients; they called this procedure the Whipple at the splenic artery (WATSA). Despite its similarity to the present RPD, it should be acknowledged that WATSA involves the artery‐last approach, with the SMA dissected after division of the body of pancreas and the splenic vein at the level of splenic artery, by which time the operation has passed the point of no return. Although the quality of perioperative imaging studies has improved recently, it remains common that intraoperative discovery of unexpected arterial involvement renders the tumour unresectable. In the present RPD, the SMA is dissected before pancreatic division, and when the SMA is found to be involved by tumour, the resection can be aborted at that point, rather than proceeding to resection with a grossly positive margin. This issue is of particular importance with modern chemotherapy, whereby more patients with borderline resectable or unresectable locally advanced PDAC are now potential candidates for resection after preoperative chemotherapy or radiotherapy. In addition, the assessment of tumour extension around the mesenteric vessels becomes difficult and inaccurate after preoperative therapy^31^, and Hackert and co‐workers^32^ recommended evaluation by intraoperative frozen sectioning at the sites around critical arteries to determine resectability before committing to morbid radical resection.

The authors believe that complete clearance of the entire retropancreatic soft tissue (fat) *en bloc* with the PV/SMV is the most critical oncological element for RPD. The first reason for this is the poor accuracy in assessing the extent of tumour infiltration around the PV. Wang *et al*.^33^ studied microscopic tumour invasion in patients who underwent PD with PVR and reported that histological invasion into the vascular wall was seen in only 67 per cent of patients, despite careful preoperative evaluation by high‐quality CT. At the area near the tumour infiltration, the perivascular soft tissue becomes fibrotic, making it difficult to distinguish by finger palpation whether the tissue is involved by cancer. Thus, skeletonization of the retropancreatic PV/SMV is required by dissecting off the perivascular soft tissue, increasing the risk of violating the tumour plane without knowledge of the degree of microscopic tumour infiltration, and leaving tissue behind with residual tumour cells. Peripancreatic fat invasion is not uncommon in resected PDAC specimens. Barenboim and colleagues^34^ reported a high incidence of peripancreatic fat invasion among patients with borderline resectable PDAC: 98 per cent in patients who had upfront resection compared with 52 per cent in those who underwent resection preceded by neoadjuvant chemotherapy. Similarly, the present authors reported previously^36^ that 77 per cent of distal pancreatic cancers resected *en bloc* with retroperitoneal fat showed microscopic retroperitoneal fat infiltration.

Despite the scale and novelty of this study, there were some limitations. First, the study design of a retrospective single institutional review incurs inherited selection bias. Although the plan for PVR was always made before surgery based on CT findings, the choice of RPD *versus* SPD with PVR was deferred to the surgeon based on their experience and preference. The initial RPD was performed in 2008, and the number of RPD procedures increased slowly, probably because of the learning curve for each surgeon; in recent years, particularly after 2015 when the new neoadjuvant therapy protocol was introduced, RPD has been performed more commonly than SPD with PVR. Furthermore, although no definitive selection criteria were set for the two procedures, surgeons preferred RPD over SPD for some advanced tumours, as R0 resection was not possible otherwise. As a result, tumour characteristics and use of neoadjuvant therapy were different in the two groups, and thus the comparison of outcomes needs to be interpreted with caution. Nevertheless, because more patients with complex tumours, such as borderline resectable or unresectable locally advanced disease, were included in the RPD group than in the SPD group, the present observations for comparable perioperative outcomes across the groups were sufficient to prove the safety and feasibility of RPD. As most of the cohort patients were treated before 2015, when the management protocol was changed for advanced PDAC, perioperative management in this cohort was somewhat outdated. In current practice, patients with borderline resectable PDAC would receive neoadjuvant therapy with multiagent systemic chemotherapy before definitive resection. Furthermore, the reported margin status in this study was also based on the traditional R definition, in which R0 was not defined by the margin length (more than 1 mm)^35^, but simply by the absence of cancer cells on each margin (margin greater than 0 mm).

RPD characterized by *en bloc* retropancreatic PV/SMV resection with surrounding soft tissue is safe and feasible, and might also improve the long‐term outcomes. A prospective randomized trial to evaluate the role of RPD is warranted in the era of modern multidisciplinary management strategy for PDAC.

## Supporting information


**Video S1** Representative video of the regional pancreatoduodenectomy procedure
